# Exponential Sensitivity and its Cost in Quantum Physics

**DOI:** 10.1038/srep20076

**Published:** 2016-02-10

**Authors:** András Gilyén, Tamás Kiss, Igor Jex

**Affiliations:** 1 Institute for Solid State Physics and Optics, Wigner Research Centre for Physics, Hungarian Academy of Sciences, Budapest, H-1121, Hungary; 2 Department of Theoretical Physics, Budapest University of Technology and Economics, Budapest, H-1111, Hungary; 3 Faculty of Nuclear Sciences and Physical Engineering, Czech Technical University in Prague, Prague, 115 19 Praha 1, Czech Republic

## Abstract

State selective protocols, like entanglement purification, lead to an essentially non-linear quantum evolution, unusual in naturally occurring quantum processes. Sensitivity to initial states in quantum systems, stemming from such non-linear dynamics, is a promising perspective for applications. Here we demonstrate that chaotic behaviour is a rather generic feature in state selective protocols: exponential sensitivity can exist for all initial states in an experimentally realisable optical scheme. Moreover, any complex rational polynomial map, including the example of the Mandelbrot set, can be directly realised. In state selective protocols, one needs an ensemble of initial states, the size of which decreases with each iteration. We prove that exponential sensitivity to initial states in any quantum system has to be related to downsizing the initial ensemble also exponentially. Our results show that magnifying initial differences of quantum states (a Schrödinger microscope) is possible; however, there is a strict bound on the number of copies needed.

Quantum technology progresses at a fast pace. Preparation, control and measurement of coherent quantum systems^1^ became possible on an unprecedented level leading to a wealth of proposals of applications ranging from quantum information processing to high precision measurements and sensors. In these protocols, increasingly sophisticated sequences of coherent evolution, measurement and post-selection are applied in order to control the state of quantum systems. Dynamics achieved by state selective protocols was proven essential for a large number of quantum information protocols^2^,^3^ and quantum communication^4^. Prominent examples of probabilistic protocols are the KLM scheme^2^ for linear optical quantum gates or the entanglement purification protocols^5^,^6^,^7^ employing measurement and selection in order to increase the entanglement between subsystems.

Manipulation by measurement and selection breaks the linearity of quantum mechanics, thereby broadening the possibilities for quantum evolution^8^,^9^,^10^,^11^. In contrast, in the well established field of quantum chaos^12^ one studies the signatures of chaos in closed quantum systems with linear evolution. In such systems the linearity of time evolution prevents distance growing between two initial quantum states. However, the essential non-linearity of an iterated, state selective protocol can result in truly chaotic behaviour^13^,^14^,^15^, meaning that initially close quantum states can get separated rapidly. The existence of such sensitive quantum protocols has been shown in^13^, but sensitivity was proved only for a tiny fractal subset of initial states having zero measure. In this article we demonstrate that exponential sensitivity can exist for all initial states in an experimentally realisable optical scheme. Moreover, we show that any complex rational polynomial map, including the example of the Mandelbrot maps^16^, can be directly realised using state selective protocols, bringing a whole new class of quantum protocols to life.

From a fundamental point of view, one can search for the most general evolution for a quantum system. A very general dynamics is sometimes imagined as the action of both unitary evolution and non-selective measurements on a system together with one or more ancillas. The evolution reduced for the system only is called a quantum channel. When talking about quantum states in practice, it is unavoidable to be able to repeat experiments on an ensemble of identically prepared initial states in order to uncover the underlying probabilistic laws. This ensemble view of quantum states allows for the following trick when designing the most general dynamics for a given initial state. Let us, for example, consider systems from the ensemble pairwise and let them interact with each other. After the interaction one can perform a measurement on one of the pairs and then discard the measured member of the pair. In case of selective measurement, one may also discard the unmeasured member of the pair, depending on the measurement result. The resulting ensemble will be reduced in size, but some of its properties may be changed in a beneficial way, e.g. entanglement between subsystems. The above procedure goes beyond the usual notion of quantum channels in the following sense. The initial step of the procedure, namely taking the systems pairwise, can be viewed as splitting the original ensemble into two parts and employing one part as an ancilla. In other words, the state of the ancilla will be dependent on the state of the system. The state dependent ancilla lies at the heart of the non-linearity of the process.

## Results

### A linear optical experimental scheme implementing a family of non-linear maps

We propose here a simple experimental setup that implements a non-linear process exhibiting exponential sensitivity to the initial state. Our scheme is inspired by an experimentally tractable entanglement purification protocol^17^,^18^ and uses only linear optical elements. At the beginning of each iterative step we form pairs of photonic qubits from the ensemble of identically prepared photons and apply a post-selective transformation on the pairs. After measuring the polarization of one photon we either keep or discard the other photon depending on the measurement result. The post-selection induces a non-linear, deterministic transformation on the remaining photons; therefore, the kept photons remain identically prepared.

**Figure 1:**
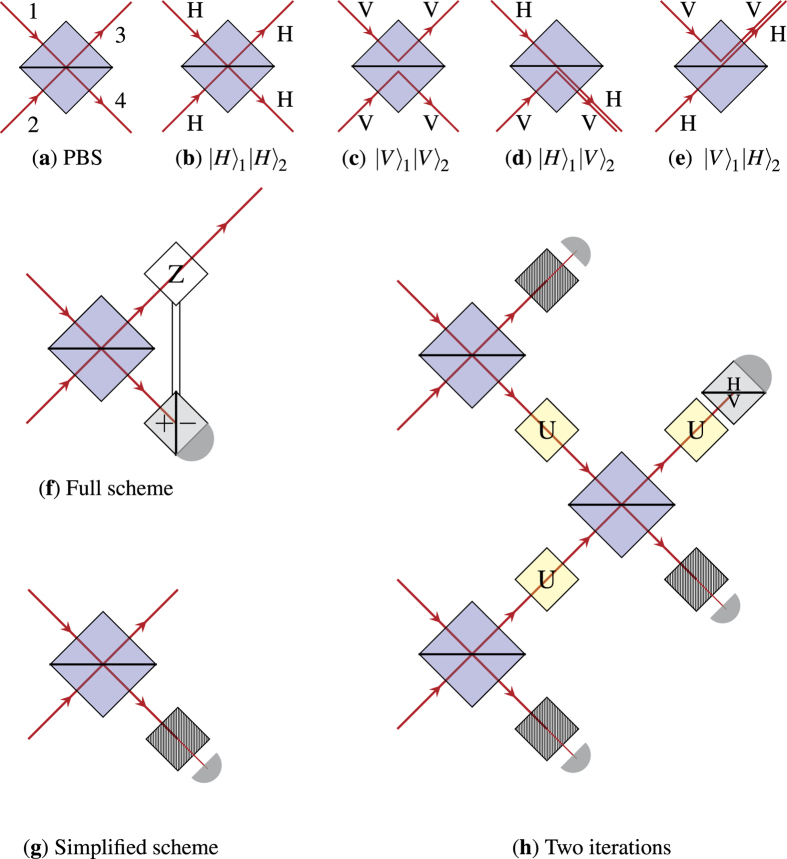
The proposed experimental setup. (**a**) A Polarizing beam splitter (PBS) with two spatial input/output modes. (**b**–**e**) The effect of the PBS acting on the four possible two photon input states regarding polarizations. (**f**) A post-selective linear optical scheme inducing a non-linear transformation (**g**) and its simplified version utilising a polarizer reducing the success probability by 1/2. (**h**) A two level scheme amended by a unitary transformation *U* acting on the polarization state of the photons. We consider a run of this experimental setup successful if all the detectors click. This condition introduces the post-selection to the system.

The key element of our scheme is the polarizing beam splitter (PBS). When two photons arrive at the same time but from different spatial input modes, this linear optical element introduces entanglement between the spatial modes and the polarization degrees of freedom, see Fig. 1. We apply post-selection and accept the output of the PBS only if there is a photon in both spatial output modes.

Let 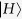 and 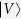 denote the horizontal and vertical polarization states for our photonic qubits. Consider the effect of the PBS acting on a product state of two incoming photons:


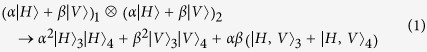


After post-selection the remaining quantum state is


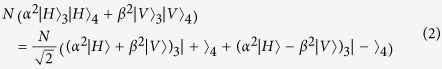


where 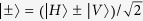 and 
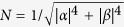
 is a norming factor. The success probability of the protocol is 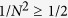. If we measure the photon at output mode 4 in the basis {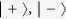}, then the other photon collapses to 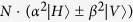 corresponding to the measurement result. To get a definite outcome 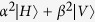 we may apply a Pauli-Z gate whenever we measure 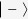 (Fig. 1(f)) or simply neglect such cases, introducing another level of post-selection (Fig. 1(g)). Either way the protocol implements a non-linear transformation 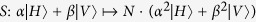 which maps the identical qubit states of an ensemble to another qubit state of a smaller identical ensemble. If we iterate this process *S* amended with an additional unitary step *U*, the iterates (*US*)^*n*^ exhibit increasingly rich dynamics.

It has been shown^13^ that by iteratively applying *US* on an ensemble of identically prepared qubits, the one qubit state of the ensemble after *n* iterations 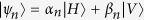 may evolve sensitively. By sensitivity we mean that for some initially similar quantum states 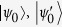 the evolving states 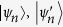 can get very different during iteration, i.e. using some quantum information distance *d* (e.g. the Bures distance) we can get 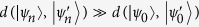. More precisely we call the process sensitive at some initial state 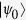 if arbitrarily close quantum states can get separated from it to a constant distance *C*, i.e. 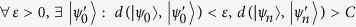. We call this exponential sensitivity if it also holds requiring 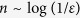.

**Figure 2:**
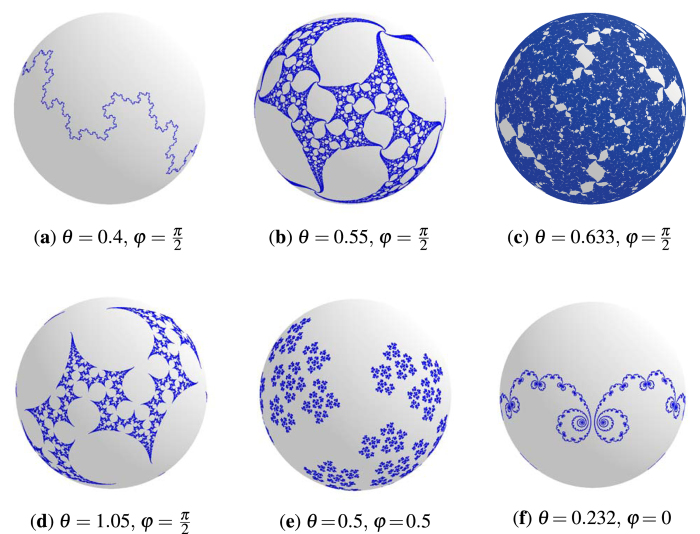
Julia sets consisting of the sensitive initial states lying on the Bloch sphere. Julia sets (blue) are plotted for various choices of 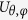. (**a**–**c**) Shows how the Julia set starts covering the whole Bloch sphere while increasing the value of *θ*. (**d**) Transition from a web to a simple closed curve. (**e**) The sensitive initial states form a completely disconnected set. (**f**) The result of a parabolic explosion (implosion)^30^ where a stable fixpoint has become unstable - breaking a circle like connected Julia set into infinitely many parts.

Sensitivity has been shown for initial states lying on a fractal subset of the Bloch sphere called the Julia set^16^,^19^, see Fig. 2. The specific fractals that were examined regarding the protocol *US*^13^ all had zero measure. However certain choices of unitaries from the family 
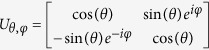
 seem to produce increasingly saturated Julia set images (see Fig. 2) suggesting that it may reach a point where the whole Bloch sphere is covered by sensitive initial states. A candidate for such a transformation is 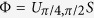, where 
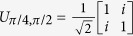
.

### Exponential sensitivity for all initial states

In order to handle the arising non-linear maps better we project the surface of the Bloch sphere to the complex plane using stereographic projection. Thus a (photonic) qubit 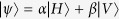 may be described using a single complex parameter including infinity: 
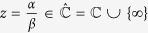
. This representation yields (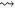) a new description of our protocol in terms of rational functions^16^,^19^:


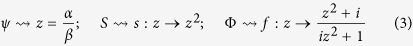


**Figure 3:**
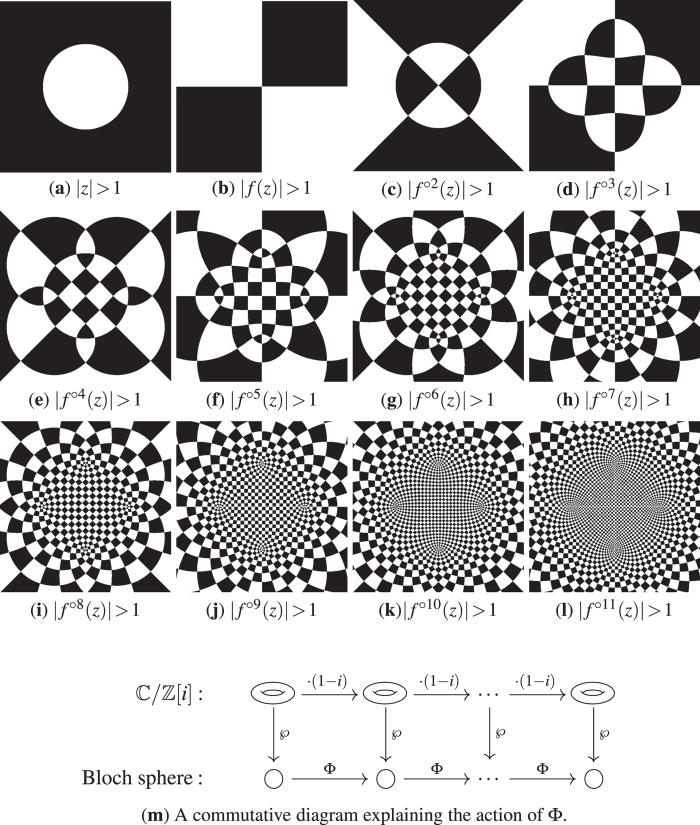
Iterations of an exponentially mixing map. (**a**–**l**) Visualisation of the iteratives of *f*. Each subfigure shows 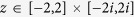; the domains are coloured according to whether 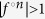
(black) or ≤1 (white) distinguishing the northern and southern half of the Bloch sphere. After a few iterations even very close states get mapped to different halves of the Bloch sphere as indicated by the rapid alternation of black and white domains. (**m**) The iterative map 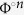 acts on the Bloch sphere correspondingly to the action of multiplication by (1 − *i*)^*n*^ on the torus, explaining the regular pattern.

Using this formalism, it turns out that *f* is one of a few special so-called Lattès maps^20^ and as such has gained a lot of attention in the theory of complex dynamical systems^19^. We can better understand the special properties of our Lattès map by analysing its relationship to the corresponding linear transformation of the 2 dimensional torus. We will represent the torus 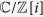 as the complex plane modulo the Gaussian integers 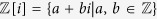. Its transformation is represented by multiplication with 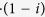: 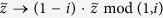, which rotates and folds the torus 2 times over itself. The correspondence between the torus and the sphere is established via the so called Weierstrass elliptic function^21^ 
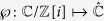
. Relating the two surfaces gives rise to the identity 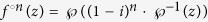 showing that iterating *f* on 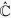 has essentially the same effect as repeatedly applying multiplication 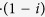 on 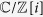, see Fig. 3 and Methods.

Viewing Φ through these glasses it becomes clear that it shows chaotic behaviour on the whole Bloch sphere. The map representing Φ on the torus uniformly stretches the surface of the torus by a factor of 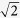 and folds over itself two times. It is intuitively clear that the iterative application of such a transformation shows exponential sensitivity to the initial position on the torus and has a positive Lyapunov exponent. Even more strikingly, it exhibits exponential mixing, yielding that during the iteration of Φ even a tiny uncertainty about the initial state evolves exponentially fast to a complete uncertainty, meaning that the iterated state may be any point of the Bloch sphere, as depicted on Fig. 3. For a rigorous derivation of the exponential mixing see Methods.

### Emergence of general complex rational dyanmics and the Mandelbrot set

We may find even more exotic transformations by generalising the protocol to allow the physical realisation of any rational map 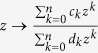 of degree *n* ≥ 2. Our generalised scheme proceeds by forming *n*-tuples of identical pure qubits 
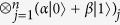
 and applying an appropriate *n*-qubit unitary *V*. The final step of one iteration is measuring all the qubits except the last one and keeping it only if all measurements resulted 0; this post-selective step can be shortly described by the projection 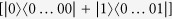. Implementing a specific rational map reduces to finding a *V* unitary satisfying:


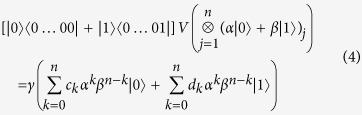


with arbitrary γ ≠ 0, providing the desired post-selected state. The existence of such *V* follows from a simple linear algebraic argument explicated in Methods.

**Figure 4:**
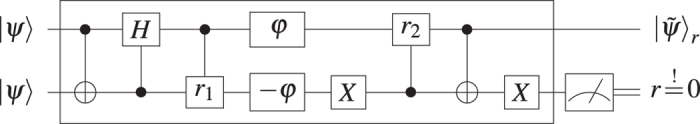
A quantum circuit implementing Mandelbrot maps. The controlled gate labelled by the real number *r*_*j*_ is essentially a rotation composed with a Pauli-*Z*-gate 
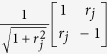
 and the gate labelled by ±*φ* is a phase gate 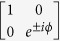, while *H* and *X* stand for the Hadamard and Pauli-*X* gates correspondingly. If we set *r*_1_ = 1/*r*_2_, 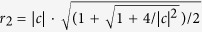 and 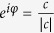, then the resulting map is *z*^2^ + *c*, provided that we accept only the 0 measurement outcome on the second qubit.

A notable consequence is that we found a direct quantum physical realisation of the Mandelbrot maps 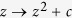 and can devise a quantum circuit for it. Figure 4 shows a possible quantum circuit implementation for this family of maps. The scheme demonstrates how the corresponding family of 2-qubit unitaries may be constructed using only controlled 1-qubit gates, which are considered experimentally more feasible in general.

For the sake of completeness we note that recently another strong connection between rational functions and quantum computing with post-selection was discovered^22^ from an algorithmic perspective.

### The cost of non-linearity and the Schrödinger microscope

We have just shown that using post-selection one can implement a wide range of non-linear maps, which may be useful for various tasks. A highly non-linear map, like Φ, provides a sort of “Schrödinger microscope”^23^ enabling one to exponentially magnify tiny differences between quantum states. Consider for example the behavior of Φ around the fixed state 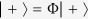. Using our representation in terms of rational functions this fixed point becomes 1 = *f* (1). Then 
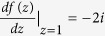
 translates to that Φ doubles infinitesimal distances around 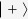, analogously to a microscope.

Having such a tool we are tempted to develop powerful quantum algorithms utilising it. It is well known that introducing post-selection to Quantum Computing makes it extremely powerful - the corresponding complexity class PostBQP^24^ includes NP and even PP. The question of efficiency and resource needs naturally arises. We address it using a black box argument considering results about state discrimination^25^,^26^,^27^.

Suppose we have a quantum device implementing *n* iterations of Φ processing a qubit ensemble of size *N*. The size *M* of the successfully processed output ensemble may be probabilistic. We would like to determine its success rate, i.e. the average ratio *M*/*N*. To derive a bound on the success rate consider applying this quantum device to the qubit ensemble having state either 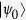 or 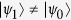 with equal probability 1/2. If their distance 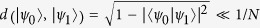, then the distance of the full ensemble states is 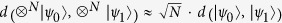. Thus we cannot distinguish the possible ensemble states with error probability less than 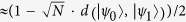, see [26, Chapter IV §2]. Suppose 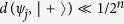 for *j* = 0, 1 then, after *n* iterations of our process Φ, the distance of the two states increases by a factor of roughly 2^*n*^ since Φ doubles infinitesimal distances around the fixed point 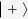. If the device outputs *M* copies of the transformed states then we can distinguish the ensembles with error probability 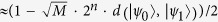. For large *N* the success rate is roughly constant because of the law of large numbers; thus we can treat the value *M*/*N* fixed. But 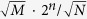 cannot exceed 1 as the error probability of discrimination cannot decrease and so the success rate is upper bounded by 4^−*n*^. This holds for states lying close to 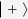, in better cases the rate may be higher. For our implementation scheme each iteration has a success rate at least 1/4 (up to a negligible term −1/*N* due to parity) implying that this scheme provides the best possible worst case success rate.

In this way we have shown that exponentially many copies are needed for *n* iterations of the process. Similar upper bound can be devised to any non-linear map that have a region where the separation of close states can be described by a multiplicative factor *λ* > 1. If we follow the above argument it turns out that the worst case success rate of such protocol is bounded by 1/*λ*^2^. Note that the particular choice of metric by which we measured separation does not limit the scope of the argument too much – we could use any other metric that agrees infinitesimally, e.g. the Bures metric. Thus it turns out that the implementation of any kind of Schrödinger microscope needs exponentially many copies of the states in terms of magnification steps (more precisely, quadratically many in terms of the total magnification).

## Discussion

While exploring the possible dynamical properties of state selective protocols, we found that any complex rational map can be implemented using state selection. Such a general and natural correspondence between a physical system and the theory of complex dynamical systems is unique to our knowledge. We could also devise a realistic optical experimental scheme, which implements particularly interesting quadratic rational dynamics.

We showed that a specific setting of the proposed optical scheme implements an exponentially mixing map. At several regions of the Bloch sphere this protocol magnifies initial differences between quantum states almost uniformly, thus we may call it a Schrödinger microscope. The term Schrödinger microscope was introduced by Lloyd and Slotine^23^ in connection with a non-linear quantum protocol emerging from collective weak measurements and coherent feedback. Even though^23^ introduces Schrödinger microscope at a conceptual level, there was no explicit example shown unlike in this article. Although the collective weak measurement approach seems very different from our post-selective scheme, they are connected at a deep level: the effective non-linearity comes from the underlying ensemble of identical quantum states in both cases.

During our study of the emergence of exponential sensitivity, we were led to the analysis of implementation cost, which turned out to be exponential. We found a general bound on the number of copies needed for the successful implementation of any expanding non-linear map. We proved that a protocol capable of magnifying differences between close quantum states by a factor *λ* > 1 necessarily has a worst case rate of loss at least 1 − 1/*λ*^2^ in the number of copies of the unknown input quantum states. This “Quantum magnification bound” is basically another reformulation of the fact that one cannot bootstrap quantum information without an external source, somewhat resembling the quantum no-cloning theorem.

We used the “Quantum magnification bound” principle to show the optimality of our implementation of a Schrödinger microscope. In general, this principle helps to understand the advantages and limitations of any kind of Schrödinger microscope, regardless of the actual implementation method. Thus it also overcomes the difficulties coming from approximative arguments required to describe complex systems, like the protocol^23^ involving collective weak measurements and coherent feedback. This fundamental bound may be applied to other quantum information protocols, providing a general tool for bounding the success rate of particular probabilistic protocols.

Looking at general processes, with inspiration coming from this principle, may also provide a new insight to the relation of classical and quantum chaos^28^, suggesting that classical deterministic chaos may be just an approximation with a characteristic time scale. Classical deterministic chaotic systems explode quickly, and observing the deterministic evolution of the system, even at a macroscopic level, enables the determination of the initial conditions increasingly precisely^29^. But there is a level of precision that is prohibited by quantum uncertainty relations. This is an apparent philosophical contradiction, provided we believe classical physics is based on quantum mechanics; a possible dissolution is saying that on long time scales one cannot treat a classical process deterministically chaotic, just chaotic in some statistical sense.

## Methods

### Connection to Lattès maps

The map 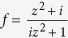 can be written as 
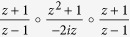
, where 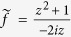 is a well-known Lattès map^20^, and 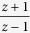 is a self inverse Möbius transformation^19^. Since conjugation by Möbius transformation does not change the iterative features, it implies that *f* exhibits the same dynamics as 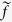. To give a physical meaning to this Möbius transformation, we mention that it corresponds to a rotation of the Bloch sphere, i.e. *f* and 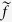 essentially describe the same process, just written in a different qubits basis.

As we already indicated in this article, *f* is conjugate to the map 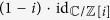 via the Weierstrass-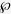 function. In fact, we need a slightly transformed version of the Weierstrass-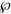 that is amended by our Möbius transformation. Our transformed version can be written as 
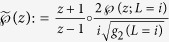
 (for the definitions of *L, g*_2_ and 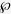 (*z*; *L*) see^21^). Using this function we get the key identity 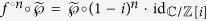.


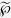
 induces a two sheet branched covering with 4 exceptional points that are covered only once. (These points can be easily spotted on Fig. 3). With the exception of these 4 points, 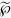 is a two-to-one map so it does not have a well defined inverse. A general point 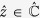 has pre-images 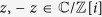, but the linear map on 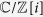 carries opposite numbers to opposite ones so fortunately the identity 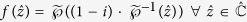 holds regardless which branch of 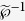 is considered. In this sense we can say, that the stronger identity 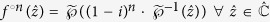 also holds.

### Metric on the Bloch sphere 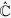 and the torus 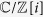


We would like to show exponential mixing of our Lattès map, thus we need to understand how distances are distorted by 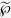. In order to trace the problem, we need to introduce some proper distance concepts.

A possible metric on pure quantum states is given by using the distance defined by the quantum angle 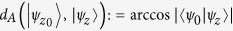. Note that this distance coincides with the natural spherical metric of the Bloch sphere, up to a multiplicative factor of 2. This metric is similar to the Bures metric defined by the distance 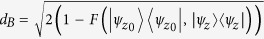, where *F*(.,.) is the Fidelity of two density matrices. A third possible distance definition is given by 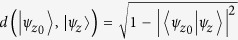. Since all three distance definitions coincide for infinitesimal distances, we are free to chose the most appropriate one for our calculations. For now we stick with the natural metric of 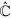, which is 2 times the quantum angle 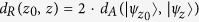 (the index *R* refers to the fact that *d*_*R*_ is a Riemannian metric). For the torus 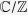 [*i*]we use the natural Riemannian metric inherited from 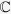.

First, we show that the Möbius transformation 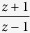 leaves the metric on 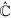 invariant. Putting it differently, we would like to show that the conformal metric 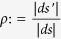 is trivial, where *ds* and *ds*′ are tangent vectors of 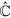 at points *s* and 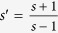 such that *ds* is mapped to *ds*′ by the tangent map. We proceed using the identity 
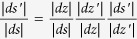
, where *dz* and *dz*′ are tangent vectors of 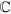. The conformal metric transformation introduced by the stereographic projection is well known to be 
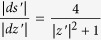
, similarly 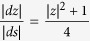. Finally 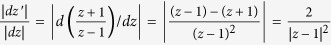. Putting everything together 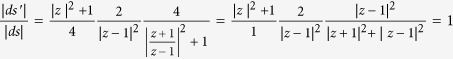 as we indicated.

The Möbius transformation is just an isometry of 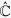, so we can concentrate on the other part 
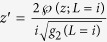
 of our Weierstrass function 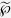. From now on let 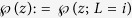, 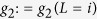 and 
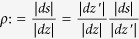
, where *dz*′ is a tangent vector of 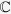 at a point *z*′. Just as above 
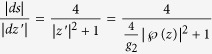
. For the other factor 
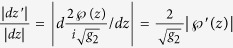
. Now we use the well known property 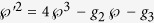. Since 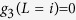 the final formula is: 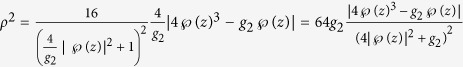. Using the triangle inequality we get 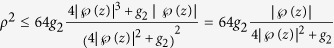. This function has its maximum when 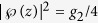; substitution yields 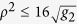. Finally using that 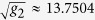, we arrive at the conclusion *ρ* < 16.

The conformal metric *ρ* < 16 is upper bounded, so the image of any two points from the surface of the torus gets mapped to points having spherical distance less than 16 times their torical distance. Thus for any point *s* on the surface of the Bloch sphere and a radius *ε* ball 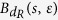 around it, the ball’s pre-image 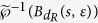 contains another ball 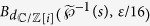.

### Exponential mixing

Suppose we have a one qubit state 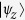 that we know with ambiguity *ε*. In other words it is a pure quantum state 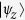 close to some 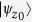, such that the quantum angle 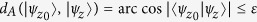. So 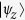 lies in the diameter *ε* ball around 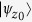, i.e. 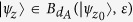. This ball corresponds to 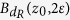 using the (Bloch) spherical representation. As we already discussed 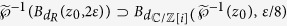.

It is easy to show that after multiplying each point of 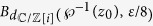 with (1 − *i*)^*n*^ the image covers the whole 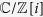, provided that 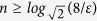. Thus for 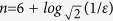 we have 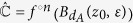. It means that after 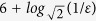 iterations the initial uncertainty about the state 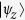 evolves so much that 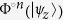 may be any pure state. This statement is basically a translation of the fact that the linear map 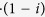 has Lyapunov exponent 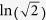 on 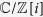, and shows exponential mixing and sensitivity on the surface of the Bloch sphere.

### Construction of *n*-qubit unitaries for degree *n* rational maps

We would like to implement the rational function 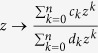. Our generalised protocol starts by forming *n*-tuples of identical pure qubits of our ensemble, then continues by the application of a specific *n*-qubit unitary *V*. The final step is a measurement on all the qubits except the last one of every tuple. The protocol succeeds if all the measurements resulted in 0, the unmeasured qubit is kept only in such cases.

Initially the state of the *n*-tuples is the following product state:


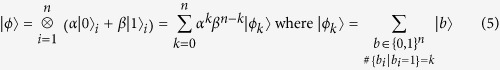


As before we use the parametrisation *z* = *α*/*β* for a qubit 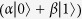. Then the parameter of the unmeasured, post-selected qubit can be described as follows:


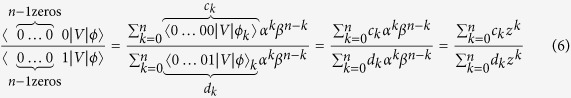


We present a linear algebraic argument showing that for any rational map of degree ≥2 there is a suitable unitary *V*. We show how to construct a unitary for any coefficients *c*_*k*_, *d*_*k*_ describing such a rational map.

First let us introduce some vector 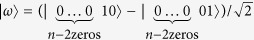 that is orthogonal to all the 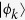 vectors. We set 
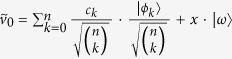
 and 
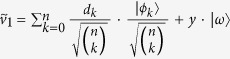
. Then by choosing 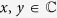 appropriately, we can always satisfy the equalities:


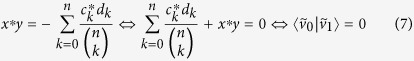



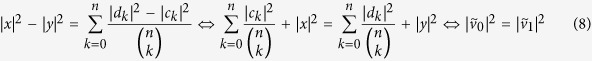


Finally, setting 
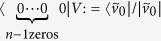
 and 
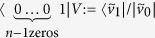
 satisfies (6). Due to (7),(8) 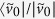 and 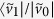 are orthonormal vectors so we can extend *V* to a full *n*-qubit unitary by defining the remaining 2^*n*^ − 2 orthonormal rows arbitrarily.

Without loss of generality we can assume that 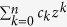 and 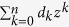 has no common roots; otherwise we can cancel it. So the probability that the process succeeds 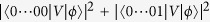 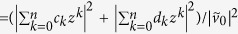 is non-zero. Then this success probability is also greater than some *p* probability for a fixed map, regardless the state 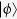 due to compactness of the state space. (However, depending on the map, this lower bound may be arbitrarily low.)

Thus using the above defined *V* unitary we can implement the rational function 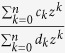 where as before this means a transformation


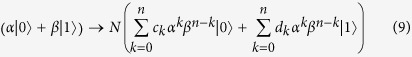


where *z* = *α*/*β* and *N* is a norming factor.

## Additional Information

**How to cite this article**: Gilyén, A. *et al*. Exponential Sensitivity and its Cost in Quantum Physics. *Sci. Rep.* **6**, 20076; doi: 10.1038/srep20076 (2016).
